# Supplementary figures and images for: Evidence for pre-climacteric activation of AOX transcription during cold-induced conditioning to ripen in European pear (Pyrus communis L.)
Source: PLoS One. 2019 Dec 4;14(12):e0225886. doi: 10.1371/journal.pone.0225886 (PMC6892529; doi:10.1371/journal.pone.0225886)

## Slide 1
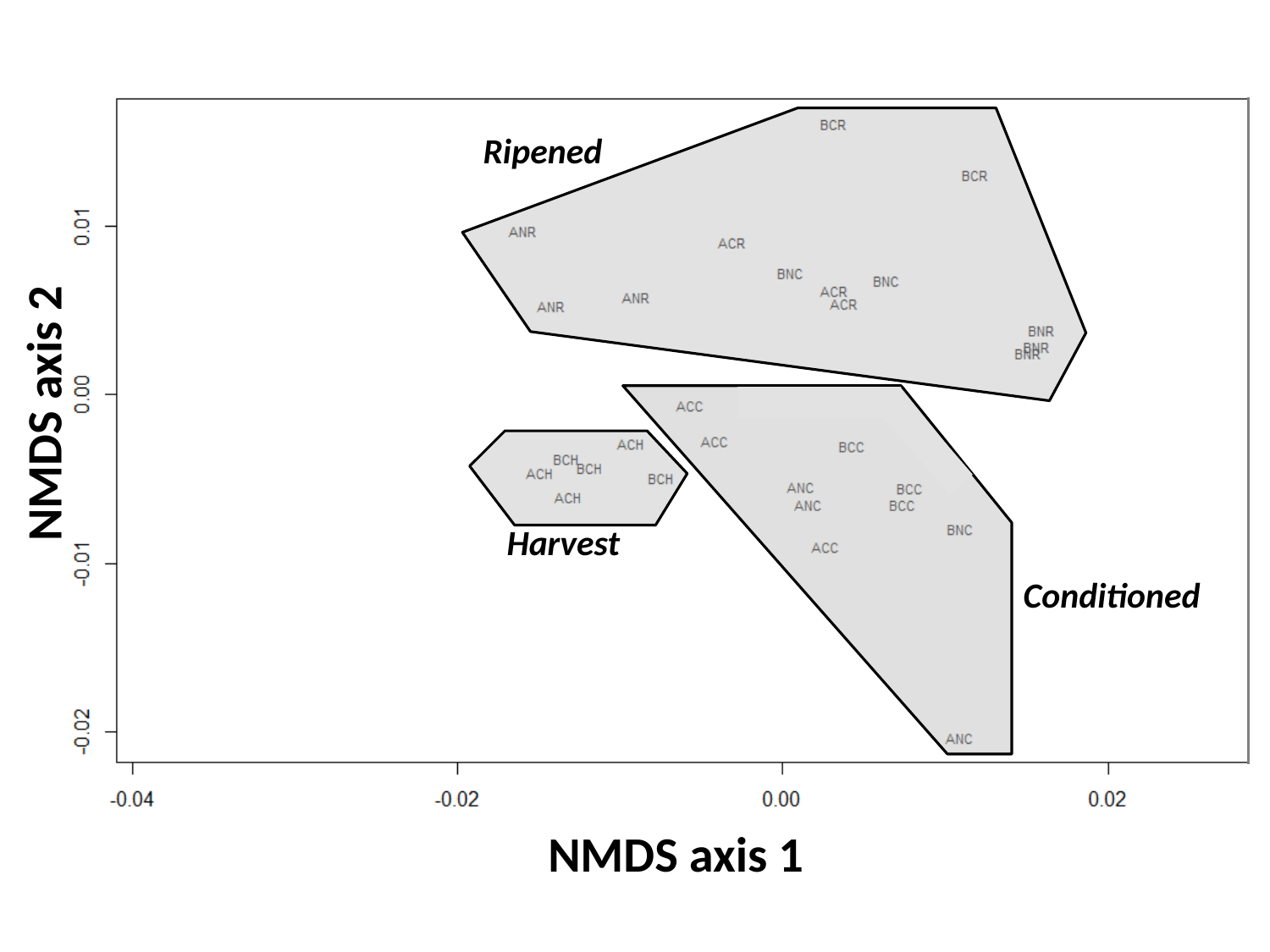

Ripened
NMDS axis 2
Harvest
Conditioned
NMDS axis 1

Supplement: S5 File — (PPTX) [file pone.0225886.s005.pptx]

## Slide 1
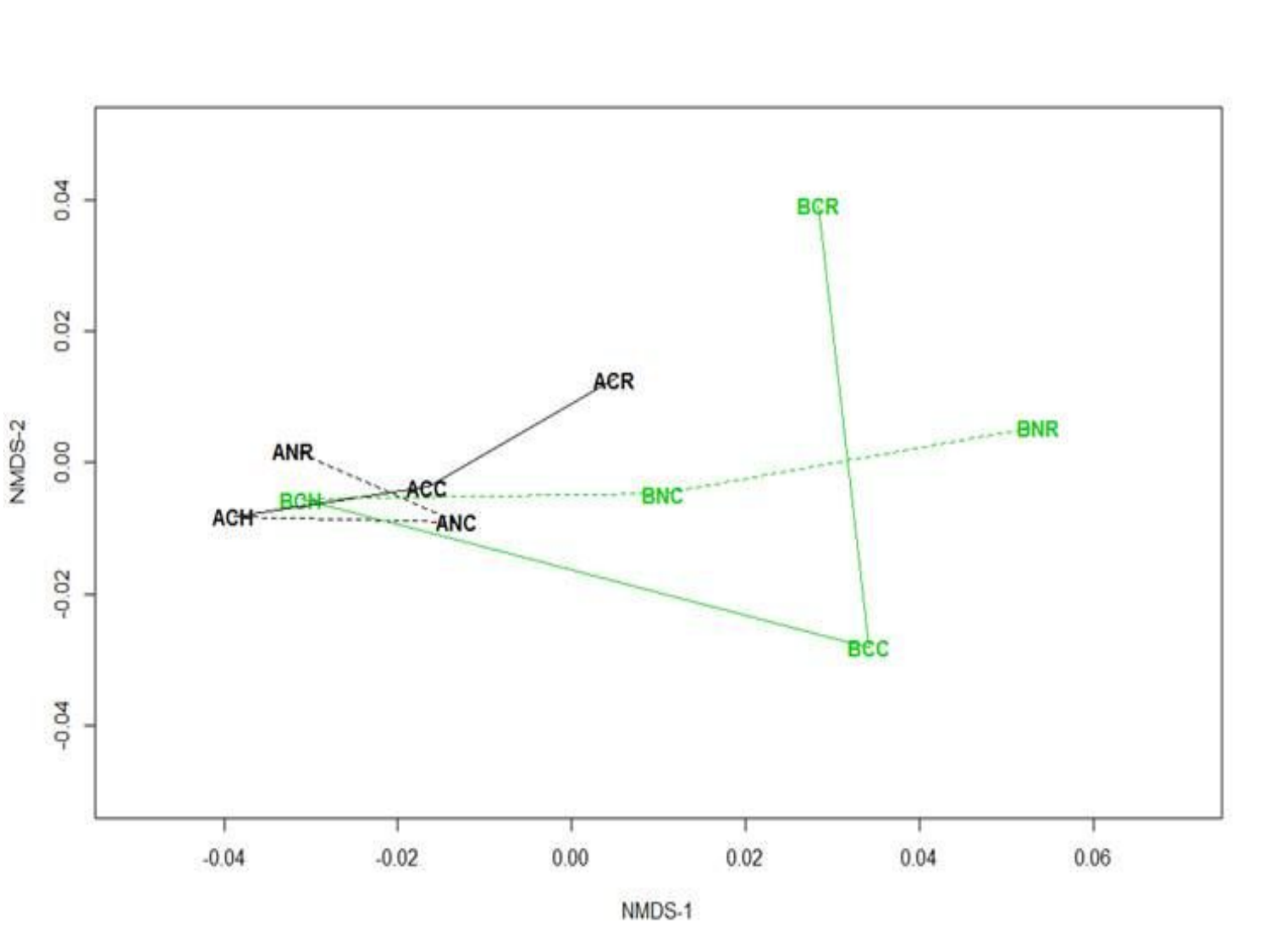

NMDS axis 2
NMDS axis 1
0.04
0.04
0.00
-0.02
-0.04
-0.04
-0.02
0.00
0.02
0.04

Supplement: S11 File — (PPTX) [file pone.0225886.s011.pptx]

## Slide 1
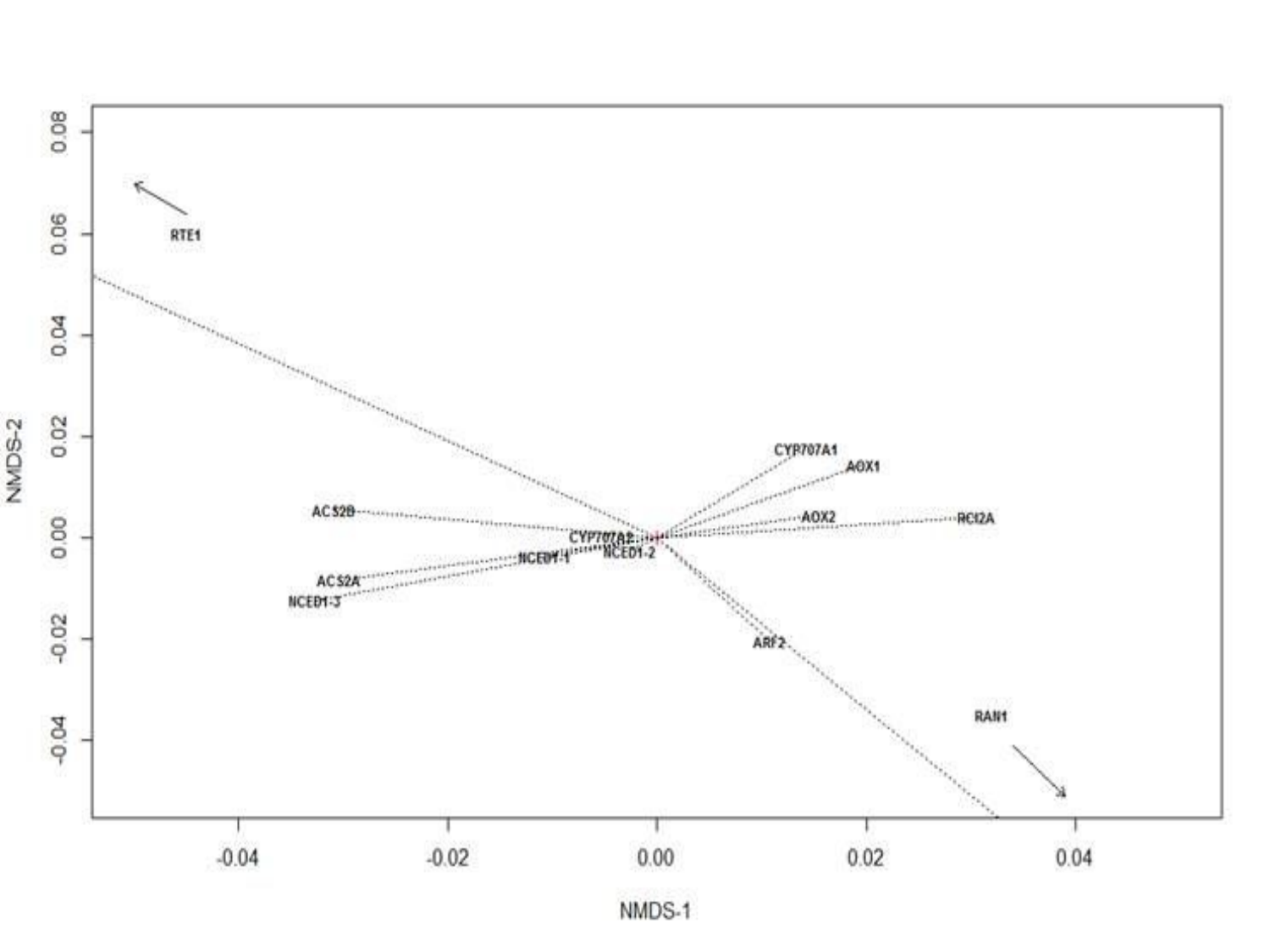

Supplement: S12 File — (PPTX) [file pone.0225886.s012.pptx]

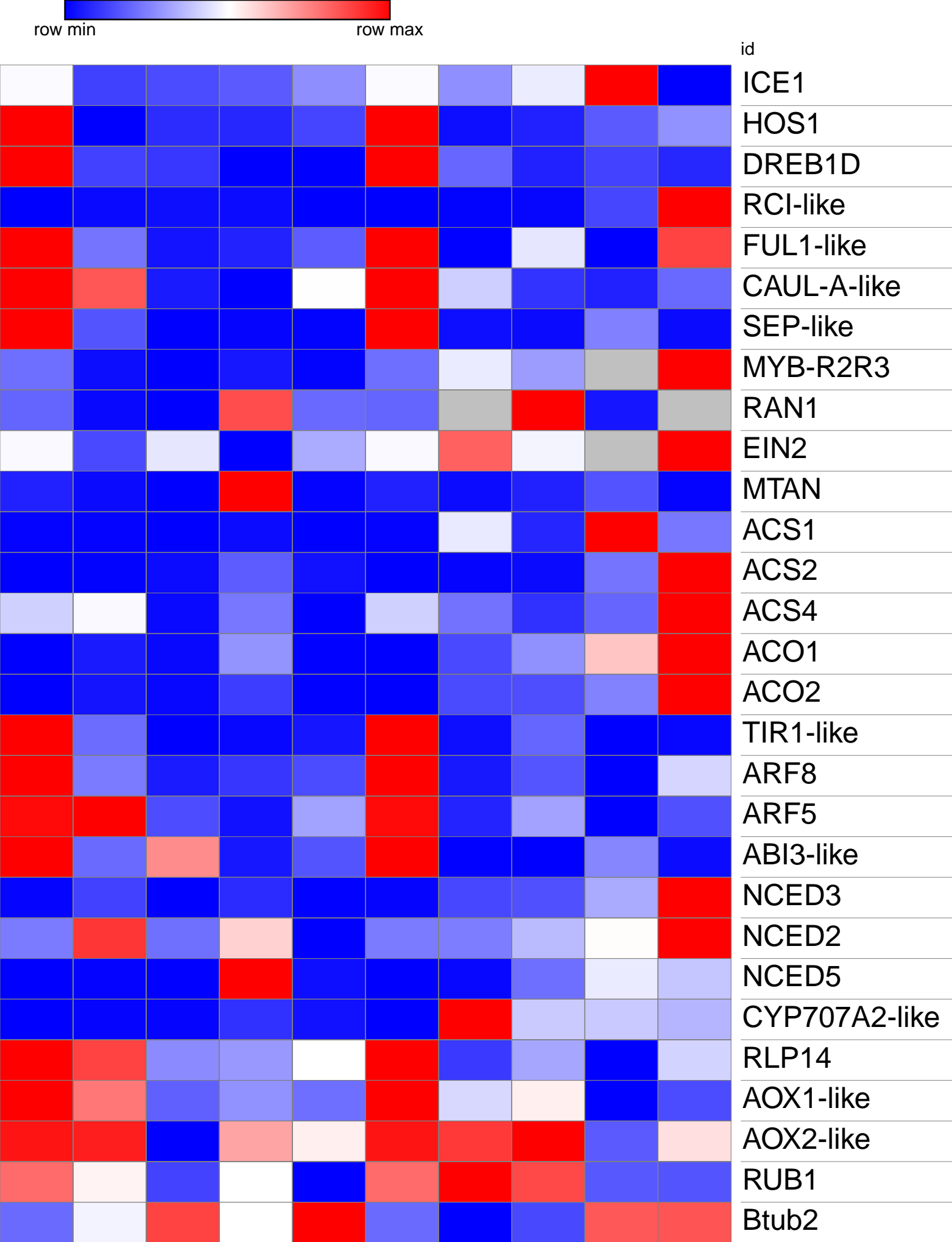

Supplement: S13 File — (PDF) [file pone.0225886.s013.pdf]
