# Supplementary material for: Evidence for pre-climacteric activation of AOX transcription during cold-induced conditioning to ripen in European pear (Pyrus communis L.)
Source: PLoS One. 2019 Dec 4;14(12):e0225886. doi: 10.1371/journal.pone.0225886 (PMC6892529; doi:10.1371/journal.pone.0225886)

**Supplemental File 8**. (A, top) Stress plots of initial (circles) and second (triangles) NMDS ordination procedures. Both instances produced a final stress coefficient of nearly 0.20 after 20 iterations. *-One gene target (VAS1) replicated (technical replicates) 3 times only. (B, bottom) Dissimilarity plot from ordination, with non-metric correlation and linear fit.


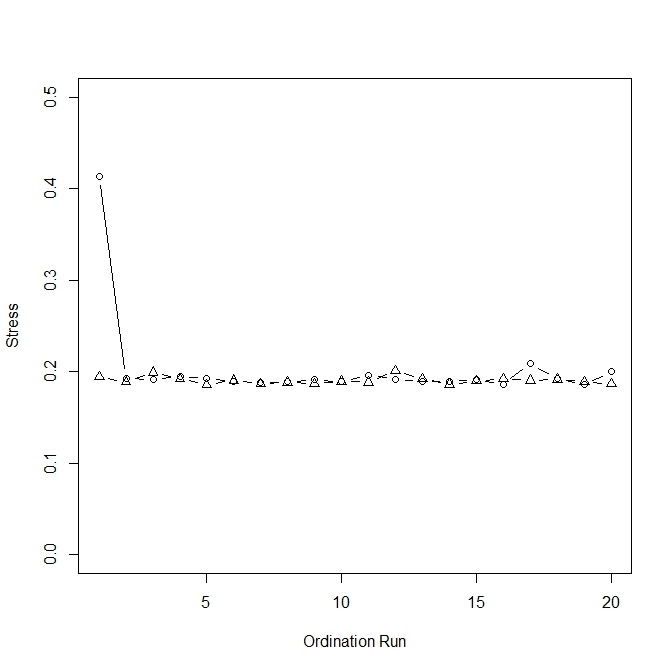


NMDS1 – 92 gene targets, 1 technical replicate

NMDS2 – 36 gene targets, 4 technical replicates*

NMDS


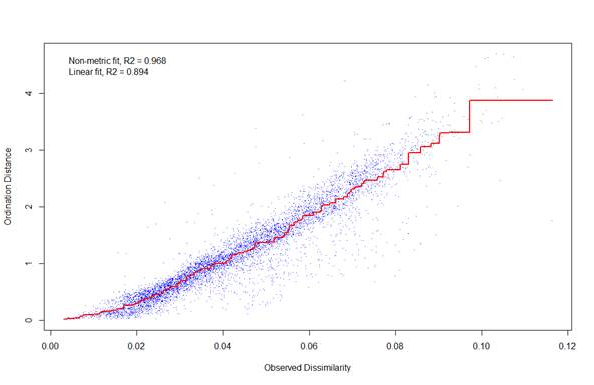

Supplement: S8 File — Stress plots of the initial (circles) and second (triangles) NMDS ordination procedures. Both instances produced a final stress coefficient of nearly 0.20 after 20 iterations. (DOCX) [file pone.0225886.s008.docx]
